# Evaluation of Multidetector Computed Tomography in the Diagnosis of Intestinal Obstruction

**DOI:** 10.7759/cureus.66244

**Published:** 2024-08-05

**Authors:** Anuradha Kelkar, Vishav Bir S Thakur, Jacob Jeeson

**Affiliations:** 1 Department of Radiology, Dr. D. Y. Patil Medical College, Hospital and Research Centre, Dr. D. Y. Patil Vidyapeeth (Deemed to be University), Pune, IND

**Keywords:** multidetector computed tomography (mdct), intestinal obstruction, cect abdomen, abdomen pain, small bowel obstruction

## Abstract

Background

Acute intestinal obstruction is a commonly encountered surgical emergency that is prevalent worldwide and has substantial morbidity and fatality rates. Therefore, swift and precise diagnosis is essential. While mortality rates in urban areas have declined due to timely medical intervention following early detection, the situation contrasts starkly in rural areas. Delays in presentations often lead to complications because of hesitancy toward surgery, economic challenges, and limited access to healthcare information. Therefore, this study aimed to evaluate how well multidetector computed tomography (MDCT) can help determine the site, cause, and level of intestinal obstruction compared to what the surgeons confirmed intraoperatively.

Methodology

A prospective study involving 101 patients was conducted at a tertiary care center in western Maharashtra from July 2022 to July 2024. The emergency department referred patients with clinical symptoms such as nausea and vomiting, abdominal distension, abdominal pain, inability to pass flatus, constipation, or diarrhea, which are commonly seen with intestinal obstruction. The study involved all patients who underwent a contrast-enhanced MDCT scan using both intravenous and oral contrast agents. We selected patients from both genders, regardless of their age; however, considerations were taken to include characteristics convenient and relevant to the study. Patients with abnormal serum creatinine levels or allergies to contrast were excluded from the study. We conducted CT examinations, noting findings such as the transition point between the dilated and collapsed loops, mesenteric fat stranding, and intestinal dilatation. An experienced radiologist made the final report, and the operating surgeons’ notes on laparoscopy or open surgery for the same patient were reviewed to understand the operative findings.

Results

MDCT scans had high diagnostic accuracy for small and large bowel obstruction. Of the 101 patients, the mean age was 43.7 years. There were 70 (69.30%) males and 31 (30.69%) females. Sensitivity was 100%, specificity was 98.1%, positive predictive value was 83.7%, and negative predictive value was 100%.

Conclusions

MDCT demonstrated high sensitivity and specificity for diagnosing and determining the underlying cause of intestinal obstruction. It identified the location of the obstruction and discerned whether it originated from intrinsic, extrinsic, or intraluminal factors.

## Introduction

The abdomen is often referred to as a “mystery box” due to its wide range of potential causes for a single clinical symptom. Intestinal obstruction is a significant concern among these possibilities. There is a wide range of symptoms associated with intestinal obstruction, from modest abdominal discomfort and bloating to severe cases that require emergency surgical intervention and even shock [1]. Similarly, intestinal obstruction is one of the most common emergencies in general surgery, contributing significantly to medical expenses and morbidity worldwide [2]. In developing and underdeveloped nations, the most prevalent causes of intestinal obstruction are mass lesions, infectious diseases, and obstructed hernias, while post-surgical adhesions are the most prevalent cause in developed nations [3].

Various mechanisms and causes contribute to intestinal obstruction. External factors include adhesions, strangulation, hernias, and extrinsic masses such as lymphoma and carcinoid tumors. Intrinsic causes include intramural hemorrhage, Crohn’s disease, radiation enteropathy, intussusception, tuberculosis, and adenocarcinoma. Intraluminal factors include intestinal malrotation and bezoars. Large bowel obstructions can also stem from carcinoma, sigmoid volvulus, cecal volvulus, and fecal impaction [4].

Early detection of intestinal obstruction poses challenges, and radiologists play a pivotal role in clinical decision-making. They provide crucial information to address specific questions that greatly influence patient management strategies [2,4]. Conventional radiography serves as the initial imaging modality for patients suspected of bowel obstruction, demonstrating an accuracy rate ranging from 46% to 80% in confirming the presence of obstruction [5]. A dilated small bowel loop measuring over 2.5 cm in diameter and more than 10 cm in length on ultrasonography indicates intestinal obstruction. Although ultrasonography can occasionally pinpoint the cause, its accuracy tends to be lower compared to computed tomography (CT). The use of diluted barium as a luminal contrast agent aids in the localization and characterization of the obstructed segment, distinguishing between complete and incomplete obstructions [6].

Unlike oral contrast radiography, which primarily visualizes the luminal surface, CT scanning offers the capability to image abdominal structures beyond the lumen. This advantage allows for a detailed assessment of the obstruction’s nature, particularly when it arises from an extraluminal or intramural malignant condition. Furthermore, CT can reveal additional abdominal pathologies, such as lymph node or liver metastases, abnormalities in solid organ parenchyma, and ascites. This thorough assessment aids in the precise identification of the root cause of the obstruction [6].

Intravenous contrast is useful for the diagnosis of pathologies including superior mesenteric vein thrombosis and occlusion, which can cause ileus that resembles mechanical obstruction, as well as for detecting strangulation and determining the precise etiology of small intestinal obstruction [7]. Contrast-enhanced abdominal CT has become the primary imaging modality due to its widespread availability, rapid image acquisition, and comprehensive assessment capabilities that aid in ruling out other potential causes of symptoms. Multidetector CT (MDCT) has a high specificity and sensitivity of approximately 95% for diagnosing high-grade bowel obstruction. However, its accuracy is relatively low in cases of partial obstruction [8,9].

Similar to radiography, the key feature in imaging for diagnosing bowel obstruction is the presence of dilated bowel loops. Specific dimensions indicating dilation include 2.5 cm for the ileum, 3 cm for the jejunum, greater than 9 cm for the cecum, and more than 6 cm for the rest of the colon. Additionally, there is typically decompression of distal bowel loops beyond the obstructed segment. These findings are characteristic hallmarks used to identify and evaluate bowel obstruction in imaging studies [10]. CT imaging offers a thorough evaluation of the bowel wall, its associated blood vessels, and the surrounding mesentery. This capability is crucial for detecting any concurrent ischemic or infarcted areas [8,9]. CT imaging is also valuable for assessing the presence of bowel perforation and detecting free extraluminal gas, which are indicative of a perforated bowel. In cases where obstruction is due to neoplastic conditions, MDCT helps identify the primary organ of origin, assess the extent of the lesion, determine its relationship with adjacent structures, and identify metastatic lesions in other organs [10].

This study aimed to optimize the diagnosis of patients who present to healthcare providers with clinical symptoms in coherence with intestinal obstruction. Early and accurate diagnosis of intestinal obstruction reduces needless exploratory laparotomies and extended hospital stays.

## Materials and methods

This observational study was undertaken among 101 patients who were referred to the radiology department at Dr. D. Y. Patil Medical College, Hospital, and Research Centre in Pimpri, Pune, between July 2022 and July 2024. These individuals were suspected of experiencing intestinal obstruction and were sent to the radiology department due to abdominal symptoms such as abdominal discomfort, bloating, nausea and vomiting, diarrhea, or constipation. The study encompassed all patients who underwent an MDCT scan utilizing both oral and intravenous contrast. The study excluded participants with high serum creatinine levels and those who had an allergic response to contrast. The cause of intestinal obstruction was determined by a CT scan and then confirmed through surgical intervention (laparoscopy or open surgery). An experienced radiologist, who had no knowledge of the surgical results, examined the MDCT scans to determine the cause of the intestinal obstruction.

All patients were called after fasting for at least six hours before the scan. After addressing the likelihood of a contrast response, each patient was asked to sign a consent form. Oral contrast (30 mL of Gastro-Video diluted in 1 L of water) was given 45 minutes before the scan. The intravenous contrast used was a 50 mL injection of Omni-Paque 350 at a rate of 3 mL/second using a pressure injector via a 20-G angiocath placed in the antecubital vein. A non-enhanced CT scan of the abdomen was performed using 5 mm thin axial sections, spaced at 5 mm intervals from the dome of the diaphragm to the pubic symphysis level, and reformatted to produce 1 mm thin sections. During the scan, the patients were instructed to hold their breath.

The possible causes of obstruction were classified into the following three categories: extrinsic, intrinsic, and intraluminal. Lesions were classified as extrinsic if their epicenter was located outside the gut, leading to a mass effect and producing intestinal obstruction. Intrinsic factors included intestinal pathologies such as infections, inflammation, or other conditions that lead to bowel obstruction. Intraluminal causes were defined as intestinal obstructions caused by masses within the bowel lumen, resulting in the complete closure of the gut lumen. The location of the intestinal obstruction was also assessed.

We acquired the data on the presence or absence of obstruction, the degree of obstruction, and the underlying cause of obstruction for CT scans by comparing it with the final diagnosis in each individual instance. The data analysis was performed via Microsoft Excel (Microsoft Corp., Redmond, WA, USA) and evaluated with SPSS Version 16 (SPSS Inc., Chicago, IL, USA). We presented the qualitative data in terms of frequency and the quantitative data in terms of means or percentages.

## Results

This study included 101 patients. Demographic findings showed that the age of the patients ranged from 7 to 82 years, with a mean of 43.5 years. Overall, 22% of the patients were in the 41-50-year age group, followed by 17% in the 0-20-year age group, as seen in Table 1. The study showed a prevalence of intestinal obstruction in 70 (69.31%) males in comparison to 31 (30.69%) females (Table 1).

**Table 1:** Age and gender correlation.

Age	Total (%)	Males (%)	Females (%)
0–20	18 (17.82%)	14 (13.86%)	4 (3.96%)
21–30	6 (5.94%)	4 (3.96%)	2 (1.98%)
31–40	16 (15.84%)	12 (11.88%)	4 (3.96%)
41–50	23 (22.77%)	15 (14.85%)	8 (7.92%)
51–60	14 (13.86%)	10 (9.90%)	4 (3.96%)
61–70	16 (15.84%)	12 (11.88%)	4 (3.96%)
71–80	6 (5.94%)	2 (1.98%)	4 (3.96%)
81–90	2 (1.98%)	1 (0.99%)	1 (0.99%)

The most frequent abdominal symptoms were abdominal pain (51.5%), nausea and vomiting (17.82%), and abdominal distension or bloating (14.85%), followed by constipation or diarrhea (12.87%), as seen in Table 2.

**Table 2:** Classification of patients based on their presenting symptoms.

Clinical symptoms	Number of patients (%)
Abdomen pain	52 (51.5%)
Abdominal distension	15 (14.85%)
Constipation/Diarrhea	13 (12.87%)
Nausea/Vomiting	18 (17.82%)
Others: fever and chills	3 (2.97%)
Total	101 (100%)

Intrinsic causes were seen in 67 patients and accounted for most cases of intestinal obstruction (66.33%). Strictures were the leading intrinsic cause of bowel obstruction seen in 30 (29.7%) patients. Extrinsic causes of obstruction were seen in 24 (23.76%) patients, with adhesions being the most common cause among extrinsic causes. A foreign body was the primary cause of intraluminal obstruction (four patients with a frequency of 3.96%) (Table 3). Intrinsic causes, with stricture being the most likely reason for obstruction, could be attributed to the higher percentage of postoperative cases undergoing MDCT scans in our tertiary center.

**Table 3:** Patients categorized by the location of bowel obstruction.

Causes of bowel obstruction		Number of patients (%)
Extrinsic – 24 (23.76%)	Adhesions	11 (10.89%)
	Hernia	8 (7.92%)
	Volvulus	1 (0.99%)
	Mesenteric ischemia	3 (2.97%)
	Appendiceal perforation	1 (0.99%)
Intrinsic – 67 (66.33%)	Stricture	30 (29.7%)
	Inflammatory	20 (19.8%)
	Tuberculosis	6 (5.94%)
	Intussusception	6 (5.94%)
	Carcinoma	5 (4.95%)
Intraluminal – 8 (7.92%)	Foreign body	4 (3.96%)
	Carcinoma	3 (2.97%)
	Appendicolith/Appendicitis	1 (0.99%)
Others – 2 (1.98%)	Dilated bowel without peristalsis – paralytic ileus	2 (1.98%)
Total		101 (100%)

The sensitivity, specificity, positive predictive value, and negative predictive value of MDCT in detecting different causes of bowel obstruction are shown in Table 4.

**Table 4:** Sensitivity, specificity, positive predictive value, negative predictive value, and diagnostic accuracy of multidetector CT in detecting common causes of intestinal obstruction.

Causes of bowel obstruction	True positive	False positive	True negative	False negative	Sensitivity (%)	Specificity (%)	Positive predictive value (%)	Negative predictive value (%)	Diagnostic accuracy (%)
Adhesions	10	1	90	0	100	98.7	90.9	100	98.9
Stricture	26	4	71	0	100	94.5	86.7	100	94.5
Hernia	7	1	93	0	100	98.9	87.5	100	98.9
Inflammatory	14	6	81	0	100	93.1	70.0	96.3	93.1
Tuberculosis	5	1	95	0	100	99	83.3	100	99
Carcinoma	6	2	93	0	100	97.9	75	100	97.9
Mesenteric ischemia	3	0	98	0	100	100	100	100	100
Foreign body	4	0	97	0	100	100	100	100	100
Intussusception	6	0	95	0	100	100	100	100	100

The MDCT scan demonstrated overall high diagnostic accuracy in diagnosing intestinal obstruction, exhibiting excellent sensitivity and positive predictive value for most causes when compared with operative findings as the gold standard. Of the total 101 patients, sensitivity was 100%, specificity was 98.1%, positive predictive value was 83.7%, and negative predictive value was 100%.

## Discussion

MDCT plays a crucial role in imaging patients presenting with acute symptoms such as nausea, vomiting, abdominal distension, abdominal pain, and diarrhea or constipation, which are indicative of intestinal obstruction. MDCT confirms the diagnosis, identifies the underlying cause of obstruction, and predicts or detects subsequent complications such as peritonitis, intestinal ischemia, perforation, and necrosis [11]. Before the widespread use of CT scans, clinical examination, laboratory studies, and plain radiographs were the primary methods for preoperative diagnosis [12]. However, CT scans have revolutionized the diagnosis of bowel obstruction by effectively differentiating patients who need surgery from those who can manage conservatively, thereby significantly enhancing clinical decision-making [8]. Surgeons emphasize the importance of early diagnosis to prevent serious complications [12], and CT scans enable personalized treatment strategies based on a thorough assessment of small and large intestine involvement, obstruction causes, and associated complications [13]. The angiographic capabilities of MDCT have also been instrumental in identifying the early vascular origins of obstructions [8,9]. MDCT does not require oral contrast for imaging intestinal obstruction because dilated bowel loops naturally contain air and fluid, providing sufficient negative contrast for imaging purposes [11]. Intravenous contrast is crucial for assessing bowel viability; non-enhancement of intestinal walls indicates non-viable tissue, distinguishing reversible from irreversible ischemia or necrosis [14]. CT scans also assist in diagnosing strangulation by identifying intestinal wall enlargement and air within the gut wall and surrounding tissues, which is crucial for determining the urgency and type of surgical intervention needed [11].

While numerous studies demonstrate the effectiveness of MDCT in accurately diagnosing intestinal obstruction, most of these studies are retrospective. Therefore, to fully understand the efficacy and impact of MDCT on the diagnosis and treatment of intestinal obstruction, additional research is necessary. Our study aimed to evaluate whether MDCT is superior to standard clinical and radiographic examinations in identifying etiology, diagnosing severity, and determining treatment options, with exploratory laparotomy as a benchmark.

In our study involving 101 patients with intestinal obstruction, the mean age at presentation was approximately 43.9 years, with a predominant age group between 41 and 50 years, consistent with findings from other studies [3,15]. MDCT exhibited a sensitivity of 94% and a specificity of 96%, aligning closely with findings reported by Megibow et al. [16]. We also observed a higher prevalence of male patients compared to female patients, with a gender ratio of 2.26, similar to other studies reporting a male predominance in intestinal obstruction cases [6,17].

All patients universally reported abdominal pain, with 52% identifying it as the predominant symptom. Other common symptoms included abdominal distension (15%), constipation or diarrhea (13%), and vomiting (18%). The findings of Goyal et al. regarding consistent symptom presentation among patients with intestinal obstruction across various studies support our observations [18]. Small bowel obstruction was predominant in 75.3% of our patients, with large bowel obstruction occurring in 24.7%, consistent with the study by Sultan et al. [19]. These results underscore the higher frequency of small bowel obstruction compared to large bowel obstruction in clinical practice [19]. In our study, stricture was the most common cause of intestinal obstruction, followed by adhesions, differing from earlier studies that reported adhesions as the primary cause [20]. A larger proportion of patients with inflammatory and infective etiologies may have contributed to the higher prevalence of strictures in our study. Consistent with previous research, CT demonstrated a sensitivity of 98.3% and a specificity of 98.4% in identifying the location of the obstruction, highlighting the robustness of CT in accurately detecting intestinal obstruction [16].

In cases of uncomplicated intestinal obstruction, conservative management commonly involves nasogastric tube decompression and supportive care. Surgical intervention becomes necessary if patients show signs of vascular compromise or fail to respond to conservative measures. Thus, CT imaging plays a critical role in predicting the need for surgical intervention, identifying imaging features indicative of bowel vascular compromise promptly to optimize management and prevent complications such as bowel ischemia, necrosis, perforation, and peritonitis [11].

Key imaging and prognostic indicators of intestinal ischemia include bowel wall thickening, ascites, pneumatosis intestinalis, and the presence of portal or mesenteric venous gas. The “target sign,” characterized by a trilaminar appearance of the ischemic bowel wall due to hyperenhancement of the mucosal layer, hypodense submucosal edema, and reduced enhancement of the outer wall, is a crucial diagnostic feature. Early recognition of these CT findings facilitates timely intervention and improves patient outcomes [21].

## Conclusions

MDCT is highly accurate in diagnosing intestinal obstruction. Results from this study are consistent with other research studies, confirming that CT should be the preferred imaging modality for diagnosing intestinal obstruction, determining its causes and precise location, and predicting the presence of bowel ischemia. This capability enables radiologists to offer essential guidance to surgeons in patient management and facilitates preoperative planning for individuals presenting with intestinal obstruction.

## References

[1] Hussain F, Fareez SN, Parveen S (2015). Intestinal obstruction-a retrospective study. IOSR J Dent Med Sci.

[2] Jun L, ChangYi S (2015). Diagnostic value of plain and contrast radiography, and multi-slice computed tomography in diagnosing intestinal obstruction in different locations. Indian J Surg.

[3] Malik AM, Shah M, Pathan R, Sufi K (2010). Pattern of acute intestinal obstruction: is there a change in the underlying etiology?. Saudi J Gastroenterol.

[4] Silva AC, Pimenta M, Guimarães LS (2009). Small bowel obstruction: what to look for. Radiographics.

[5] Gore RM, Levine MS Textbook of Gastrointestinal Radiology. Gut.

[6] Saini DK, Chaudhary P, Durga CK, Saini K (2013). Role of multislice computed tomography in evaluation and management of intestinal obstruction. Clin Pract.

[7] Frager D (2002). Intestinal obstruction role of CT. Gastroenterol Clin North Am.

[8] Khurana B, Ledbetter S, McTavish J, Wiesner W, Ros PR (2002). Bowel obstruction revealed by multidetector CT. AJR Am J Roentgenol.

[9] Tawfik HG, Al Obeidi AS (2009). Acute mechanical intestinal obstruction: the problem of diagnosis of strangulation without CT scans. Qatar Med J.

[10] Morrison ID, McLaughlin P, Maher MM (2015). Current status of imaging of the gastrointestinal tract: imaging techniques and radiation issues. Grainger & Allison's Diagnostic Radiology: Abdominal Imaging.

[11] O'Malley RG, Al-Hawary MM, Kaza RK, Wasnik AP, Platt JF, Francis IR (2015). MDCT findings in small bowel obstruction: implications of the cause and presence of complications on treatment decisions. Abdom Imaging.

[12] Ramanaiah GV, Manohar K (2015). Acute intestinal obstruction in adults - its outcome a prospective study in a tertiary health care center in Andhra Pradesh. Indian J Appl Res.

[13] Paulson EK, Thompson WM (2015). Review of small-bowel obstruction: the diagnosis and when to worry. Radiology.

[14] Dhatt HS, Behr SC, Miracle A, Wang ZJ, Yeh BM (2015). Radiological evaluation of bowel ischemia. Radiol Clin North Am.

[15] Adhikari S, Hossein MZ, Das A, Mitra N, Ray U (2010). Etiology and outcome of acute intestinal obstruction: a review of 367 patients in Eastern India. Saudi J Gastroenterol.

[16] Megibow AJ, Balthazar EJ, Cho KC, Medwid SW, Birnbaum BA, Noz ME (2024). Bowel obstruction: evaluation with CT. Radiology.

[17] Singh A, Makkar IK, Thukral C (2018). Intestinal obstruction: role of MDCT with surgical correlation. Asian J Med Radiol Res.

[18] Goyal SK, Chhabra UK, Bansal SK (2016). Intestinal obstruction-a retrospective study of 150 cases. Int Arch Integr Med.

[19] Sultan A, Hassan M, Ali M (2020). Role of multidetector computed tomography with multiplanar and curved multiplanar reformations in the detection of cause of intestinal obstruction: a tertiary care experience. Cureus.

[20] Sindhwani G, Patel V, Jain A, Arora MA, Shah P (2017). Multidetector computed tomography (MDCT) in gastrointestinal obstruction: one symptom myriad differentials. Int J Anat.

[21] Sugi MD, Menias CO, Lubner MG, Bhalla S, Mellnick VM, Kwon MH, Katz DS (2018). CT findings of acute small-bowel entities. Radiographics.

